# Disease Burden and the Accumulation of Multimorbidity of Noncommunicable Diseases in a Rural Population in Henan, China: Cross-sectional Study

**DOI:** 10.2196/43381

**Published:** 2023-05-22

**Authors:** Ying Chen, Mingming Pan, Yaling He, Xiaokang Dong, Ze Hu, Jian Hou, Yining Bao, Jing Yang, Yinghao Yuchi, Ruiying Li, Linghui Zhu, Ning Kang, Wei Liao, Shuoyi Li, Chongjian Wang, Lei Zhang

**Affiliations:** 1 Department of Epidemiology and Biostatistics College of Public Health Zhengzhou University Zhengzhou, Henan China; 2 China-Australia Joint Research Center for Infectious Diseases School of Public Health Xi’an Jiaotong University Health Science Center Xi’an, Shaanxi China; 3 Artificial Intelligence and Modelling in Epidemiology Program, Melbourne Sexual Health Centre, Alfred Health Melbourne Australia; 4 Central Clinical School, Faculty of Medicine Monash University Melbourne Australia

**Keywords:** multimorbidity, prevalence, associating factors, noncommunicable disease accumulation, NCD accumulation, public health

## Abstract

**Background:**

Multimorbidity causes substantial disease and economic burdens on individuals and the health care system.

**Objective:**

This study aimed to explore the disease burden of multimorbidity and the potential correlations among chronic noncommunicable diseases (NCDs) in a rural population in Henan, China.

**Methods:**

A cross-sectional analysis was performed using the baseline survey of the Henan Rural Cohort Study. Multimorbidity was defined as the simultaneous occurrence of at least two NCDs in a participant. This study examined the multimorbidity pattern of 6 NCDs, including hypertension, dyslipidemia, type 2 diabetes mellitus, coronary heart disease, stroke, and hyperuricemia.

**Results:**

From July 2015 to September 2017, a total of 38,807 participants (aged 18-79 years; 15,354 men and 23,453 women) were included in this study. The overall population prevalence of multimorbidity was 28.1% (10,899/38,807), and the multimorbidity of hypertension and dyslipidemia was the most common (8.1%, 3153/38,807). Aging, higher BMI, and unfavorable lifestyles were significantly associated with a higher risk of multimorbidity (multinomial logistic regression, all *P*<.05). The analysis of the mean age at diagnosis suggested a cascade of interrelated NCDs and their accumulation over time. Compared with participants without 2 conditional NCDs, participants with 1 conditional NCD would have higher odds of another NCD (1.2-2.5; all *P*<.05), and those with 2 conditional NCDs would elevate the odds of the third NCD to 1.4-3.5 (binary logistic regression, all *P*<.05).

**Conclusions:**

Our findings indicate a plausible tendency for the coexistence and accumulation of NCDs in a rural population in Henan, China. Early prevention of multimorbidity is essential to reduce the NCD burden in the rural population.

## Introduction

Multimorbidity is the coexistence of multiple disease conditions over time. Many of these conditions shared similar age-associated structural, physiologic, and biological changes in predispose patients with multimorbidity, resulting in compound risks and adversities [1-4]. The occurrence of multimorbidity is often a powerful predictor of poor health outcomes in the future. Recent studies have demonstrated that people with multimorbidity were at significantly increased risks of depression, disability, and premature death. The physiological interaction between multiple disease conditions increases the difficulty of treating individual conditions [5]. Multimorbidity often leads to a substantial financial burden on individuals, families, and the health care system [6-10].

In China, this prevalence ranged from 6.4% to 76.5%, as reported in 2015 [11]. Previous studies have reported that aging, physical inactivity, and higher BMI were contributors to noncommunicable diseases (NCDs) and their multimorbidity [12]. However, as the study population and the targeted NCDs varied, the prevalence and associating factors of multimorbidity also substantially varied across different studies [11-13]. Further, in China, most studies have focused on older adults and were conducted among the community-dwelling population [14,15], whereas research on multimorbidity in rural adults remains limited. With an aging population and high levels of risk factors, multimorbidity is becoming a public health issue in China. However, understanding the trend and characteristics of the coexistence and accumulation of NCDs is limited.

The United States has emphasized the importance of timely diagnosis of multiple NCDs to support the development of effective clinical guidelines for multimorbidity [16]. In addition, previous studies found that NCDs can mutually interact with each other [17-22]. However, to our knowledge, the study of the accumulation of NCDs based on the temporal sequence of NCD onset was limited. A better understanding of the accumulation of NCDs can help explore complex interactions between NCDs and develop interventions for managing multimorbidity to diminish the public health burden.

Our study aimed to explore the disease burden of multimorbidity and determine the pattern of NCD accumulation among rural adults in Henan, China. We recruited 39,259 participants aged 18-79 years in Henan province for this study.

## Methods

### Study Population

The baseline survey of the Henan Rural Cohort Study enrolled a total of 39,259 participants aged from 18 to 79 years between July 2015 and September 2017 in the counties of Yuzhou, Xinxiang, Tongxu, Yima, and Suiping in Henan Province by using the stratified cluster sampling method. Detail description of the cohort had been previously published [23]. In this study, participants were excluded if they (1) had cancer or renal failure (n=350; for abnormal metabolism [protein, glucose, and lipid]) [24,25] or (2) did not have complete NCD information on hypertension, dyslipidemia, type 2 diabetes mellitus (T2DM), coronary heart disease (CHD), stroke, or hyperuricemia (n=102). Ultimately, 38,807 participants were included in this study (see the study flowchart about the data process in Multimedia Appendix 1).

### Ethics Approval

This study was approved by the Zhengzhou University Life Science Ethics Committee (ethics approval 2015 MEC [S128]). Informed consent was signed by all participants.

### Data Collection and Measurements

Information was collected through face-to-face interviews using a standardized questionnaire. Demographic characteristics included age (18-44, 45-59, and ≥60 years, according to the classification provided by the World Health Organization [WHO] [26]), gender, educational level (elementary school or below, junior high school, and senior high school or above), marital status (married or cohabiting and living alone), and average monthly individual income (<500 RMB [<US $72.4], 500-1000 RMB [US $72.4-144.8], and ≥1000 RMB [≥US $144.8], according to the average monthly income of Chinese rural populations [27]). Lifestyle factors included smoking status (never, former, and current), drinking status (never, former, and current), and physical activity (low, moderate, and high) [28]. Dietary status included vegetable and fruit intake (low [<500 g/day] and high [≥500 g/day]), salt diet (low and high according to dietary flavor habits), and fat diet (low [<75 g/day] and high [≥75 g/day] according to an average taking of meat from livestock and poultry). Family history of hypertension, dyslipidemia, diabetes, CHD, stroke, and gout were also recorded.

BMI was calculated as weight in kilograms divided by height in meters squared, and further categorized as underweight (<18.5 kg/m^2^), normal (18.5-23.9 kg/m^2^), overweight (24.0-27.9 kg/m^2^), and obesity (≥28.0 kg/m^2^) [29]. Systolic blood pressure and diastolic blood pressure were obtained by using an electronic sphygmomanometer (HEM-770afuzzy, Omron). Venous blood samples were obtained from individuals after at least eight hours of overnight fasting. Total cholesterol, triglyceride, high-density lipoprotein cholesterol, low-density lipoprotein cholesterol, fasting blood glucose, and serum uric acid (SUA) level were measured by a chemistry analyzer (Cobas C501, Roche Diagnostics GmbH).

### Definitions of NCDs and Multimorbidity

In light of the lack of a standard approach to measure multimorbidity, the selection of morbidities to be included is inevitably subjective and relies on the previous studies. We have selected 6 NCDs—hypertension, dyslipidemia, T2DM, CHD, stroke, and hyperuricemia—that have been reported to be the core diseases in multimorbidity studies [30,31]. These 6 NCDs have been defined in our original survey for the cohort [23]. The definition of hypertension was average systolic blood pressure ≥140 mm Hg, diastolic blood pressure ≥90 mm Hg, or self-reported hypertension diagnosed by a physician and receiving antihypertensive therapy within the past 2 weeks [32]. Dyslipidemia was defined as one of the following conditions: elevated total cholesterol level (≥6.2 mmol/L); elevated low-density lipoprotein cholesterol (≥4.1 mmol/L); low high-density lipoprotein cholesterol (<1.0 mmol/L); elevated triglyceride (≥2.3 mmol/L); or self-reported dyslipidemia diagnosed by a physician and the use of anti-dyslipidemia medications in the past 2 weeks [33]. T2DM was defined as fasting blood glucose ≥7.0 mmol/L or self-reported T2DM diagnosed by a physician and the use of antidiabetic medications in the past 2 weeks [33]. CHD and stroke were defined as self-reports of a previous diagnosis by specialist physicians according to criteria recommended by the WHO [34,35]. The definition of hyperuricemia was SUA >7.0 mg/dL (417.0 μmol/L) in men and SUA >6.0 mg/dL (357.0 μmol/L) in women. Multimorbidity was defined as the simultaneous occurrence of at least two NCDs in a single patient [36-39].

### Statistical Analysis

The participants were divided into healthy individuals, individuals living with 1 NCD, those with multimorbidities of 2 NCDs, and those with multimorbidities of ≥3 NCDs. Continuous variables were expressed as mean (SD), and ANOVA was used for statistical analysis. Categorical variables were presented as counts and percentages, and the chi-square test was used for statistical analysis.

The prevalence of multimorbidity was assessed for each characteristic group, and we quantified the number of events for each NCD separately and visualized all observed combinations of NCDs with an intersection diagram. Multinomial logistic regression analysis was used to analyze the relationship between demographic factors, lifestyle, BMI, or family history of NCDs and 1 NCD or multimorbidity. When one factor was analyzed, all other factors were adjusted as covariance.

Based on the mean age at diagnosis, the temporal order of NCDs was determined, and binary logistic regression was used to explore the associations between NCD pairs. An NCD with an earlier onset was regarded as a predictor of an NCD with a later onset [40]. Following the cascade of NCD onset, binary logistic regression was used to assess the combined association of 2 conditional NCDs with a third, later NCD by creating a new combined variable with 4 groups of both conditional NCDs, in which the combined group without both conditional NCDs served as the reference. Demographic characteristics, lifestyle factors, dietary status, BMI, and family history of NCDs were adjusted in the regression models. In addition, in the regression of assessing the combined association, when 3 of the conditions were analyzed, the others were adjusted.

Results from the logistic regression models were presented as odds ratio (OR) with a 95% CI. The level of statistical significance was set at *P*<.05. Statistical analysis was conducted using SPSS (version 21.0; IBM Corp) and R (version 4.0.4; R Foundation for Statistical Computing).

Our study was implemented according to the Strengthening of the Reporting of Observational Studies in Epidemiology (STROBE) guidelines [41] (see the study STROBE checklist for cross-sectional studies in Multimedia Appendix 2).

## Results

### Characteristics of Study Participants

Table 1 showed the characteristics of the participants of the baseline survey from July 2015 to September 2017. The mean age was 55.6 (SD 12.2) years among the 38,807 participants. Across the NCD groups, age, gender, educational level, average monthly individual income, marital status, physical activity, smoking status, drinking status, high-salt diet, high vegetable and fruit intake, high-fat diet, and BMI were all significantly different (ANOVA and chi-square test, all *P* values were <.001, except high-salt diet [*P*=.008]). Compared with the healthy individuals, participant groups with 1, 2, or 3 or more NCDs were more likely to be older and women and have lower education and income, higher BMI, poorer marital status, a higher-salt diet, and lower physical activity level (ANOVA and chi-square test, all *P*<.001). They were also less likely to have a high-fat diet and adequate fruit and vegetable intake than healthy individuals (chi-square test, all *P*<.001).

**Table 1:** Demographic characteristics of study participants according to multimorbidity.

Variables		Total participants (n=38,807)	No NCDs^a^ (n=14,918)	1 NCD (n=12,990)	2 NCDs (n=7285)	≥3 NCDs (n=3614)	*P* value
Age (year), mean (SD)		55.6 (12.2)	52.2 (12.7)	52.3 (11.7)	58.6 (11.0)	60.8 (9.9)	<.001
Gender, women, n (%)		23,453 (60.4)	9552 (64)	7623 (58.7)	4200 (57.6)	2078 (57.5)	<.001
**Educational level, n (%)**							<.001
	Elementary school or below	17,366 (44.8)	5912 (39.6)	5869 (45.2)	3592 (49.3)	1993 (55.2)	
	Junior high school	15,457 (39.8)	6408 (43)	5241 (40.3)	2643 (36.3)	1165 (32.2)	
	Senior high school or above	5984 (15.4)	2598 (17.4)	1880 (14.5)	1050 (14.4)	456 (12.6)	
Marital status, living alone, n (%)		3979 (10.3)	1335 (8.9)	1314 (10.1)	884 (12.1)	446 (12.3)	<.001
**Average monthly income, n (%)**							<.001
	<500 RMB (<US $72.4)	13,825 (35.6)	4949 (33.2)	4693 (36.1)	2705 (37.1)	1478 (40.9)	
	500-1000 RMB (US $72.4-144.8)	12,778 (32.9)	4966 (33.3)	4251 (32.7)	2482 (34.1)	1079 (29.9)	
	≥1000 RMB (≥US $144.8)	12,204 (31.5)	5003 (33.5)	4046 (31.2)	2098 (28.8)	1057 (29.2)	
**Physical activity, n (%)**							<.001
	Low	12,544 (32.3)	4126 (27.7)	4132 (31.8)	2699 (37.1)	1587 (43.9)	
	Moderate	14,631 (37.7)	5899 (39.5)	4905 (37.8)	2587 (35.5)	1240 (34.3)	
	High	11,632 (30)	4893 (32.8)	3953 (30.4)	1999 (27.4)	787 (21.8)	
**Smoking, n (%)**							<.001
	Never	28,225 (72.7)	11,180 (75)	9319 (71.7)	5147 (70.6)	2579 (71.4)	
	Former	3131 (8.1)	901 (6)	1019 (7.9)	726 (10)	485 (13.4)	
	Current	7451 (19.2)	2837 (19)	2652 (20.4)	1412 (19.4)	550 (15.2)	
**Drinking, n (%)**							<.001
	Never	29,981 (77.3)	11,860 (79.5)	9958 (76.7)	5463 (75)	2700 (74.7)	
	Former	1794 (4.6)	523 (3.5)	549 (4.2)	432 (5.9)	290 (8)	
	Current	7032 (18.1)	2535 (17)	2483 (19.1)	1390 (19.1)	624 (17.3)	
High-salt diet, n (%)		6937 (17.9)	2609 (17.5)	2319 (17.9)	1351 (18.6)	658 (18.2)	.008
More vegetable and fruit intake, n (%)		16,183 (41.7)	6541 (43.8)	5367 (41.3)	2880 (39.5)	1395 (38.6)	<.001
High-fat diet, n (%)		7421 (19.1)	3136 (21)	2447 (18.8)	1263 (17.3)	575 (15.9)	<.001
BMI (kg/m^2^), mean (SD)		24.8 (3.6)	23.51 (3.2)	25.03 (3.4)	26.2 (3.6)	27.0 (3.5)	<.001

^a^NCD: noncommunicable disease.

### Prevalence of NCDs and Their Multimorbidities

**Figure 1:**
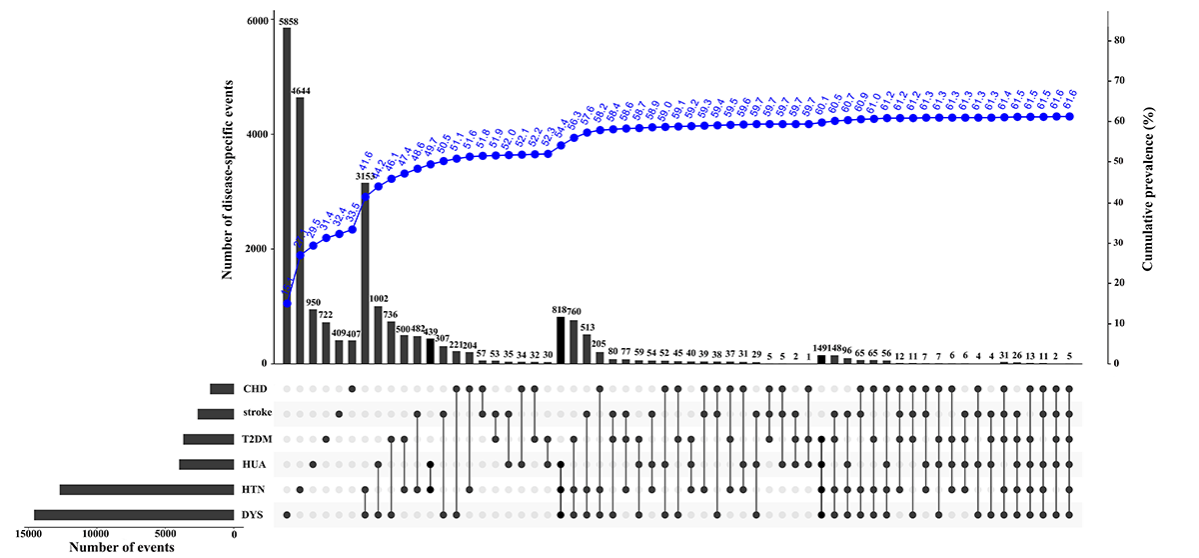
NCDs multimorbidity. The left panel displayed bars for each NCD separately that quantify the total number of events per disease, and the line chart showed the cumulative prevalence of NCDs. CHD: coronary heart disease; DYS: dyslipidemia; HTN: hypertension; HUA: hyperuricemia; NCDs: noncommunicable diseases; T2DM: type 2 diabetes mellitus.

Among all participants, 33.5% (12,990/38,807) were living with 1 NCD, among which dyslipidemia was the most common (5858/38,807, 15.1%). In contrast, 18.8% (7285/38,807) of participants were living with 2 NCDs, and the most frequent cluster of NCDs was dyslipidemia and hypertension (3153/38,807, 8.1%). Moreover, 9.3% (3614/38,807) of participants were living with 3 or more NCDs, and the cluster of dyslipidemia, hypertension, and hyperuricemia (818/38,807, 2.1%) was the most common in participants (Figure 1 and Table 2).

Overall, the prevalence of multimorbidity (2 or ≥3 NCDs) was 28.1% (10,899/38,807), and it was significantly higher in participants who were aged over 60 years (1330/3982, 36%), men (4621/15,354, 30.1%), educated in elementary school or below (5585/17,366, 32.2%), living alone (1330/3982, 33.4%), had low physical activity (4286/12,544, 34.2%), and obese (3280/6878, 47.7%; chi-square test, all *P*<.001; Table 2).

**Table 2:** The prevalence of 1 noncommunicable disease (NCD) and multimorbidity.

Variables		1 NCD, n (%)	2 NCDs, n (%)	≥3 NCDs, n (%)
Total (n=38,807)		12,990 (33.5)	7285 (18.8)	3614 (9.3)
**Age (years)**				
	18-44 (n=6805)	1963 (28.6)	728 (10.7)	222 (3.3)
	45-59 (n=15,181)	5225 (34.4)	2734 (18)	1156 (7.6)
	≥60 (n=16,821)	5802 (34.5)	3823 (22.7)	2236 (13.3)
Gender, women (n=23,453)		7623 (32.5)	4200 (17.9)	2078 (8.9)
**Educational level**				
	Elementary school or below (n=17,366)	5869 (33.8)	3592 (20.7)	1993 (11.5)
	Junior high school (n=15,457)	5241 (33.9)	2643 (17.1)	1165 (7.5)
	Senior high school or above (n=5984)	1880 (31.4)	1050 (17.6)	456 (7.6)
Marital status, living alone (n=3979)		1314 (33)	884 (22.2)	446 (11.2)
**Average monthly income**				
	<500 RMB (<US $72.4; n=13,825)	4693 (34)	2705 (19.6)	1478 (10.7)
	500-1000 RMB (US $72.4-144.8; n=12,778)	4251 (33.3)	2482 (19.4)	1079 (8.4)
	≥1000 RMB (≥US $144.8; n=12,204)	4046 (33.2)	2098 (17.2)	1057 (8.7)
**Physical activity**				
	Low (n=12,544)	4132 (32.9)	2699 (21.5)	1587 (12.7)
	Moderate (n=14,631)	4905 (33.5)	2587 (17.7)	1240 (8.5)
	High (n=11,632)	3953 (34)	1999 (17.2)	787 (6.8)
**Smoking**				
	Never (n=28,225)	9319 (33)	5147 (18.2)	2579 (9.1)
	Former (n=3131)	1019 (32.6)	726 (23.2)	485 (15.5)
	Current (n=7451)	2652 (35.6)	1412 (19)	550 (7.4)
**Drinking**				
	Never (n=29,981)	9958 (33.2)	5463 (18.2)	2700 (9)
	Former (n=1794)	549 (30.6)	432 (24.1)	290 (16.2)
	Current (n=7032)	2483 (35.3)	1390 (19.8)	624 (8.9)
High-salt diet (n=6937)		2319 (33.4)	1351 (19.5)	658 (9.5)
More vegetable and fruit intake (n=16,183)		5367 (33.2)	2880 (17.8)	1395 (8.6)
High-fat diet (n=7421)		2447 (33)	1263 (17)	575 (7.8)
**BMI**				
	Underweight (n=938)	233 (24.8)	67 (7.1)	19 (2)
	Normal (n=15,567)	4867 (31.3)	1938 (12.5)	687 (4.4)
	Overweight (n=15,306)	5546 (36.2)	3243 (21.2)	1611 (10.5)
	Obesity (n=6878)	2309 (33.6)	2012 (29.3)	1268 (18.4)
Family history of hypertension (n=7522)		2490 (33.1)	1657 (22)	1023 (13.6)
Family history of diabetes (n=1618)		523 (32.3)	339 (21)	194 (12)
Family history of CHD^a^ (n=3106)		1053 (33.9)	534 (17.2)	306 (9.9)
Family history of hyperlipemia (n=1378)		420 (30.5)	249 (18.1)	152 (11)
Family history of stroke (n=3313)		1089 (32.9)	582 (17.6)	360 (10.9)
Family history of gout (n=80)		26 (32.5)	13 (16.3)	7 (8.8)

^a^CHD: coronary heart disease.

### Associating Factors of Multimorbidity

Overall, participants with older age (all *P*<.001), those who are living alone (*P*<.001 and *P*=.007), those who are overweight and obese (all *P*<.001), former drinkers (all *P*<.001), current drinkers (*P*=.006 and *P*=.047), and those with a history of family hypertension and diabetes (all *P*<.001) were at a significantly higher risk of multimorbidity (2 or ≥3 NCDs, respectively; multinomial logistic regression). Although women (all *P*<.001) and participants with moderate and high physical activity (all *P*<.001), high-fat diet (*P*=.004 and *P*=.008), and who are underweight (all *P*<.001) were less likely to report multimorbidity (2 or ≥3 NCDs, respectively), individuals who currently smoke were less likely to report multimorbidity (≥3 NCDs; multinomial logistic regression, *P*<.001; Table 3).

**Table 3:** The associating factors of 1 noncommunicable disease (NCD) and multimorbidity.

Variables		1 NCD		2 NCDs		≥3 NCDs	
		OR^a^ (95% CI)	*P* value	OR (95% CI)	*P* value	OR (95% CI)	*P* value
**Age (years)**							
	18-44	1.0		1.0		1.0	
	45-59	1.7 (1.6-1.8)	<.001	2.5 (2.3-2.7)	<.001	3.5 (3.0-4.1)	<.001
	≥60	2.6 (2.4-2.8)	<.001	5.2 (4.7-5.8)	<.001	10.7 (9.1-12.7)	<.001
Gender, women		0.8 (0.7-0.8)	<.001	0.8 (0.7-0.8)	<.001	0.7 (0.6-0.8)	<.001
**Educational level**							
	Elementary school or below	1.0		1.0		1.0	
	Junior high school	1.0 (0.9-1.0)	.53	0.9 (0.8-0.9)	.005	0.8 (0.7-0.9)	<.001
	Senior high school or above	1.0 (0.9-1.0)	.24	1.0 (0.9-1.1)	.64	0.9 (0.8-1.0)	.08
Marital status, living alone		1.1 (1.0-1.2)	.08	1.3 (1.2-1.4)	<.001	1.2 (1.1-1.4)	.007
**Average monthly income**							
	<500 RMB (<US $72.4)	1.00		1.0		1.0	
	500-1000 RMB (US $72.4-144.8)	1.0 (0.9-1.0)	.08	1.0 (0.9-1.1)	.75	0.8 (0.7-0.9)	<.001
	≥1000 RMB (≥US $144.8)	1.0 (0.9-1.0)	.28	0.9 (0.9-1.0)	.12	1.0 (0.9-1.1)	.42
**Physical activity**							
	Low	1.0		1.0		1.0	
	Moderate	0.9 (0.8-0.9)	<.001	0.8 (0.7-0.8)	<.001	0.6 (0.6-0.7)	<.001
	High	0.8 (0.7-0.8)	<.001	0.6 (0.6-0.7)	<.001	0.4 (0.4-0.5)	<.001
**Smoking**							
	Never	1.0		1.0		1.0	
	Former	1.0 (0.9-1.1)	.52	1.1 (0.9-1.2)	.26	1.2 (1.1-1.4)	.02
	Current	1.0 (0.9-1.1)	.51	1.0 (0.9-1.1)	.62	0.8 (0.7-0.9)	<.001
**Drinking**							
	Never	1.0		1.0		1.0	
	Former	1.0 (0.9-1.2)	.75	1.4 (1.2-1.7)	<.001	1.8 (1.5-2.1)	<.001
	Current	1.1 (1.0-1.2)	.07	1.2 (1.1-1.3)	.006	1.1 (1.1-1.3)	.047
High-salt diet		1.0 (1.0-1.1)	.70	1.1 (1.0-1.1)	.26	1.0 (0.9-1.1)	.55
More vegetable and fruit intake		1.0 (0.9-1.0)	.10	1.0 (0.9-1.0)	.13	1.0 (0.9-1.1)	.43
High-fat diet		0.9 (0.9-0.9)	.03	0.9 (0.8-0.9)	.004	0.9 (0.8-0.9)	.009
**BMI**							
	Underweight	0.6 (0.5-0.7)	<.001	0.4 (0.3-0.5)	<.001	0.3 (0.2-0.5)	<.001
	Normal	1.0		1.0		1.0	
	Overweight	2.0 (1.9-2.1)	<.001	3.0 (2.8-3.2)	<.001	4.4 (4.0-4.9)	<.001
	Obesity	3.3 (3.1-3.6)	<.001	7.8 (7.1-8.6)	<.001	14.8 (13.1-16.6)	<.001
Family history of hypertension		1.4 (1.3-1.5)	<.001	1.9 (1.8-2.1)	<.001	2.7 (2.4-2.9)	<.001
Family history of diabetes		1.2 (1.1-1.3)	.04	1.4 (1.2-1.6)	<.001	1.6 (1.3-1.9)	<.001
Family history of CHD^b^		1.0 (1.0-1.1)	.41	0.9 (0.8-1.1)	.29	1.1 (0.9-1.3)	.27
Family history of hyperlipemia		0.9 (0.8-1.1)	.20	1.0 (0.8-1.2)	.77	1.1 (0.9-1.4)	.33
Family history of stroke		1.0 (0.9-1.0)	.25	0.9 (0.8-0.9)	.02	1.1 (0.9-1.2)	.36
Family history of gout		0.9 (0.5-1.5)	.61	0.8 (0.4-1.5)	.47	0.8 (0.3-1.9)	.55

^a^OR: odds ratio.

^b^CHD: coronary heart disease.

### Age Gaps Between NCDs

**Figure 2:**
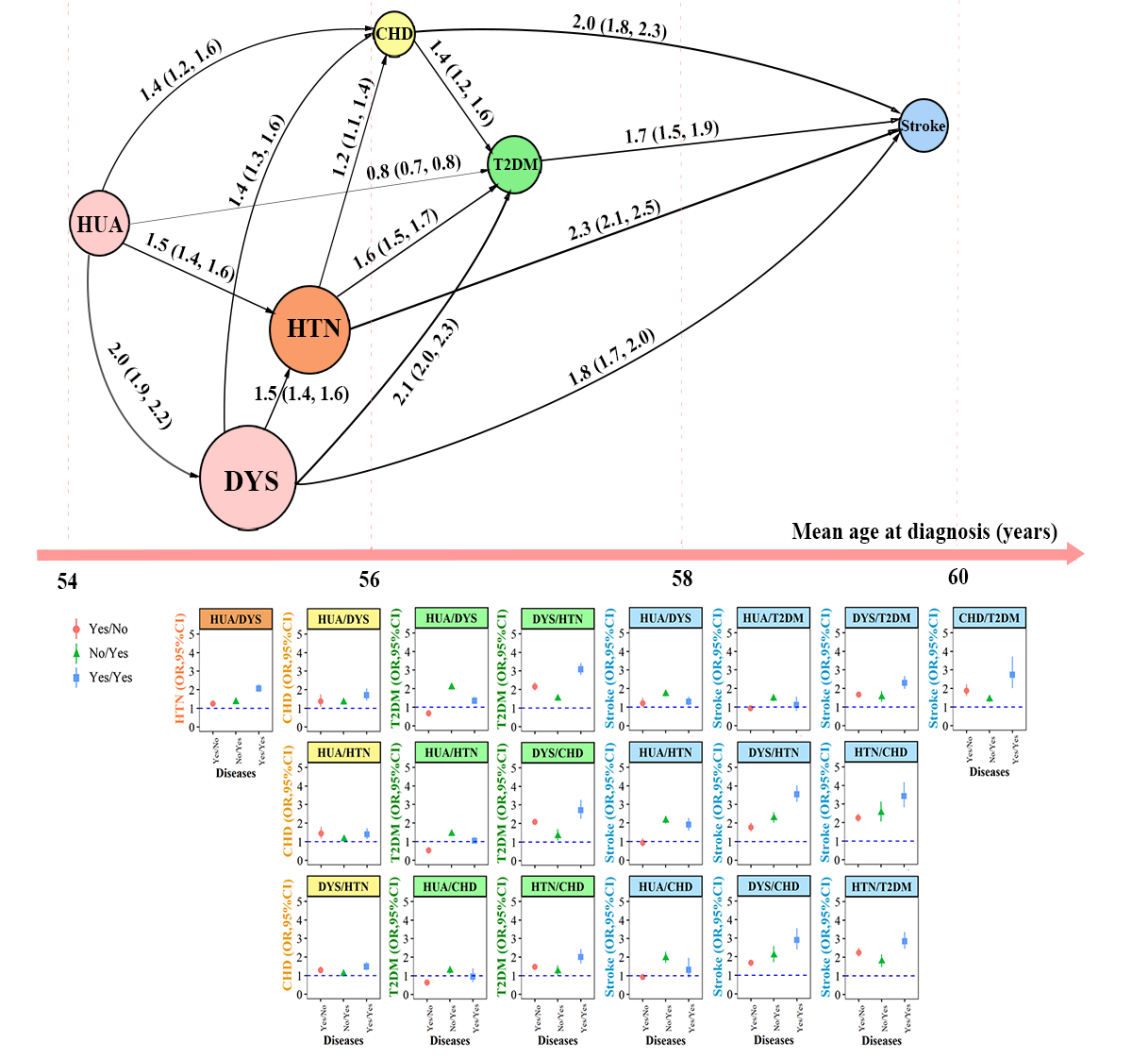
Age gaps between NCDs and the accumulation of NCDs in individuals based on mean age at diagnosis. NCDs along the x-axis were in order of increasing mean age at diagnosis. The number of the connected edge reflected the OR (95% CI) of the NCD pairs. The forest plot displayed the independent and combined effects of NCDs. The dots and lines exhibited OR and 95% CI, respectively. The model reference was "no/no," which meant without 2 conditional NCDs; "yes/no" meant having the first NCD of the 2 conditional NCDs, "no/yes" meant having the second NCD, and "yes/yes" meant having both conditional NCDs. CHD: coronary heart disease; DYS: dyslipidemia; HTN: hypertension; HUA: hyperuricemia; NCD: noncommunicable disease; OR: odds ratio; STR: stroke; T2DM: type 2 diabetes mellitus.

A cascade of NCDs was depicted by the mean age of participants at diagnosis. Among the study participants, 10.2% (3973/38,807) were diagnosed with hyperuricemia at a mean age of 54.0 years, followed by dyslipidemia (14,569/38,807, 37.5% at 55.1 years), hypertension (12,692/38,807, 32.7% at 55.4 years), CHD (1708/38,807, 4.4% at 56.1 years), T2DM (3664/38,807, 9.4% at 56.7 years), and stroke (2613/38,807, 6.7% at 59.1 years). In addition, we observed additional connections between NCD pairs (Figure 2 and Multimedia Appendix 3).

### Impact of Multimorbidity on Hypertension Occurrence

Compared with individuals without 2 conditional NCDs, individuals with 1 conditional NCD (hyperuricemia or dyslipidemia) would have significantly higher odds of hypertension (1.3-1.4, logistic regression; both *P*<.001). Individuals with 2 conditional NCDs (hyperuricemia and dyslipidemia) would further elevate their odds of hypertension to 2.1 (*P*<.001; Figure 2 and Multimedia Appendix 4).

### Impact of Multimorbidity on CHD Occurrence

Compared with individuals without 2 conditional NCDs, individuals with one of the conditional NCDs (hyperuricemia, dyslipidemia, or hypertension) would have significantly higher odds of CHD (1.2-1.5; the combination of hyperuricemia without dyslipidemia: *P*=.008; the combination of no hyperuricemia but dyslipidemia: *P*<.001; the combination of hyperuricemia without hypertension: *P*=.001; and the combination of no hyperuricemia but hypertension: *P*=.02). Individuals with 2 conditional NCDs (hyperuricemia and dyslipidemia combination: *P*<.001; or hyperuricemia and hypertension combination: *P*=.002) would further elevate their odds of CHD to 1.4-1.7 (Figure 2 and Multimedia Appendix 4).

### Impact of Multimorbidity on T2DM Occurrence

Compared with individuals without 2 conditional NCDs, individuals with 1 conditional NCDs (dyslipidemia, hypertension, or CHD) would have a significantly higher odd of T2DM (1.3-2.2; all *P* values were <.001 except the combination of no dyslipidemia but CHD [*P*=.01]), and individuals with these 2 conditional NCDs (dyslipidemia and hypertension combination or dyslipidemia and CHD combination) would further elevate their odds of T2DM to 2.7-3.1 (all *P*<.001; Figure 2 and Multimedia Appendix 4).

### Impact of Multimorbidity on Stroke Occurrence

Compared with individuals without 2 conditional NCDs, individuals with one of the conditional NCDs (dyslipidemia, hypertension, CHD, or T2DM) would have significantly higher odds of stroke (1.4-2.5; all *P*<.001). Individuals with these 2 conditional NCDs (6 NCD combinations) would further elevate their odds of a stroke to 2.3-3.5 (all *P*<.001; Figure 2 and Multimedia Appendix 4).

## Discussion

### Principal Findings

Our findings indicated a strong tendency for the coexistence and accumulation of NCDs in a rural population in Henan, China. The prevalence of multimorbidity was 28.1% (10,899/38,807) and increased with age, unfavorable lifestyles, and a higher BMI among Henan rural adults. The multimorbidity of dyslipidemia and hypertension was the most prevalent in study participants of all age groups. In addition, the onset of NCDs was associated, and a later NCD is often predictable by 1 or more preceding NCDs. The tendency of NCD accumulation indicates that compared with participants without conditional NCDs, participants with 1 existing NCD are more likely to develop a second NCD, whereas participants with 2 NCDs have an even greater likelihood to develop a third NCD.

Our study finding demonstrated a lower multimorbidity prevalence than previous studies [14]. A younger age, existing recall and reporting biases, and a fewer number of NCDs included may have contributed to the lower prevalence observed in our study. Further, consistent with previous studies [2,42,43], the prevalence of multimorbidity increases with age, and approximately one-third of older adults aged 60-79 years develop multimorbidity. Nevertheless, multimorbidity in the young and middle-aged population should not be neglected, given the increasing trend of risk factors in the young and middle-aged population [13,44]. Multimorbidity research and preventive strategies should not focus on older adults only but should instead recognize its importance throughout the whole life cycle.

Our study found that unfavorable lifestyles and a higher BMI were associated with a higher risk of multimorbidity. Our finding is supported by evidence in other settings. A multinational cohort study found that a prediagnostic healthy lifestyle, such as a healthy diet, lower BMI, and higher physical activity, could reduce the risk of multimorbidity [45]. A pooled study from 16 cohort studies demonstrates that a high BMI significantly contributes to the risk of cardiometabolic multimorbidity [46]. Further, a systematic review identifies that physical activity may improve immunity and reduce systemic inflammation and thereby ameliorate multimorbidity [47]. Overall, many modifiable risk factors associated with multimorbidity, including drinking, overweight and obesity, and physical inactivity, could be targets of behavioral prevention and interventions. Differing from previous studies [48-50], our study found that a high-fat diet, current smoking status, and being underweight may reduce the risk of multimorbidity. Given our study is a cross-sectional study with certain biases, the power to explain the causative effects of risk factors on diseases is limited. Further investigation is necessary to confirm this particular finding. Our findings support the WHO’s recommendation to implement evidence-based strategies to reduce poor use of alcohol, encourage physical activity, and maintain a healthy weight.

Our study suggested a tendency for NCD accumulation among the participants. The clustering of dyslipidemia and hypertension was the most prevalent multimorbidity observed in our study, consistent with previous studies [30,51]. A Japanese study further describes hypertension as the most common comorbidity among individuals with dyslipidemia [52]. In addition, the temporal sequence reflects the order of NCDs onset and suggests a potential causal association between the occurrence of these NCDs and the development of other comorbidities [40]. The accumulation of NCDs reflects that participants who lived with 1 conditional NCD are more likely to develop a second NCD, whereas individuals with 2 conditional NCDs are more likely to develop a third NCD than otherwise. Similar results have also been reported in a large-series prospective study: participants with the multimorbidity of T2DM and hypertension have a higher risk of CHD or stroke than participants with hypertension or T2DM [53]. This observation might be partly explained by obesity and the variations of metabolites that have accumulating side effects on health over time [54,55]. Kivimaki et al [54] found that obesity-related disease predicted or was predicted by 1 or more other obesity-related diseases, leading to the accumulation of NCDs. A study of 11,000 participants to examine metabolic pathways associated with 27 NCDs shows that two-thirds of metabolites are associated with more than 1 NCD. In addition, 420 metabolites shared between multimorbidity have been found, revealing several key common pathways in NCDs [55]. Our study suggests that taking reasonable health management and therapies of early NCDs can reduce the incidence or slow down the progression of multimorbidities effectively and ultimately improve the quality of life of the population [56,57].

Our findings have important implications for research and public health policy for multimorbidity. This study indicates that multimorbidity is an important health concern in rural Henan. In light of the WHO Sustainable Development Goals for preventing and controlling NCDs [58], existing health care needs to be improved to cope with the burden of multimorbidity. First, the prevalence of multimorbidity is higher in those older than 45 years. A life cycle approach to multimorbidity and to the challenges it poses to public health is vitally important [59]. Second, there is a clear need to increase the awareness of the importance of a healthy lifestyle in the rural population in Henan. Measures of propagating publicity and education on relevant health knowledge, improving health literacy, and changing unfavorable lifestyles should be implemented. Third, disease-specific guidelines are insufficient to effectively manage patients with multimorbidity, and new detailed guidelines for multimorbidity need to be developed [6]. Given the tendency of the coexistence and accumulation of NCDs, preventing multimorbidity will become difficult if the NCDs are treated separately. Integrated strategies for multimorbidity should be developed, such as monitoring at the individual level with electronic health records and increasing the screening management of high-risk populations.

### Limitations

This study has several limitations. First, the causal relationship between NCDs may not be reliable on account of the data being from the baseline survey of a prospective cohort study in Henan Province. Therefore, prospective studies are needed to confirm the results of this study. Second, we define hypertension, dyslipidemia, hyperuricemia, and T2DM by self-report with laboratory measurements, whereas CHD and stroke are defined only by self-report, which might underestimate their prevalence. Finally, although several potential confounders are controlled, there may be other unknown confounders affecting the results of this study.

### Conclusions

In conclusion, our study indicates that multimorbidity is an important health issue in rural Henan. Older age, unfavorable lifestyles, and a higher BMI are associated with a higher risk of multimorbidity. In addition, our findings highlight a plausible explanation for the coexistence and accumulation of NCDs. Care for multimorbidity is complex, and concerted efforts and early interventions to prevent the occurrence of multimorbidity are essential to improve health in the rural population in Henan, China.

## Appendix

Multimedia Appendix 1 Study flowchart about the data process.

Multimedia Appendix 2 The Strengthening of the Reporting of Observational Studies in Epidemiology (STROBE) statement checklist for cross-sectional studies.

Multimedia Appendix 3 The association between noncommunicable disease pairs based on mean age at diagnosis.

Multimedia Appendix 4 The accumulation of noncommunicable diseases in individuals based on mean age at diagnosis.

## Abbreviations

CHD: coronary heart disease
NCD: noncommunicable disease
OR: odds ratio
STROBE: Strengthening of the Reporting of Observational Studies in Epidemiology
SUA: serum uric acid
T2DM: type 2 diabetes mellitus
WHO: World Health Organization
