# Supplementary figures and images for: Disease Burden and the Accumulation of Multimorbidity of Noncommunicable Diseases in a Rural Population in Henan, China: Cross-sectional Study
Source: JMIR Public Health Surveill. 2023 May 22;9:e43381. doi: 10.2196/43381 (PMC10242500; doi:10.2196/43381)

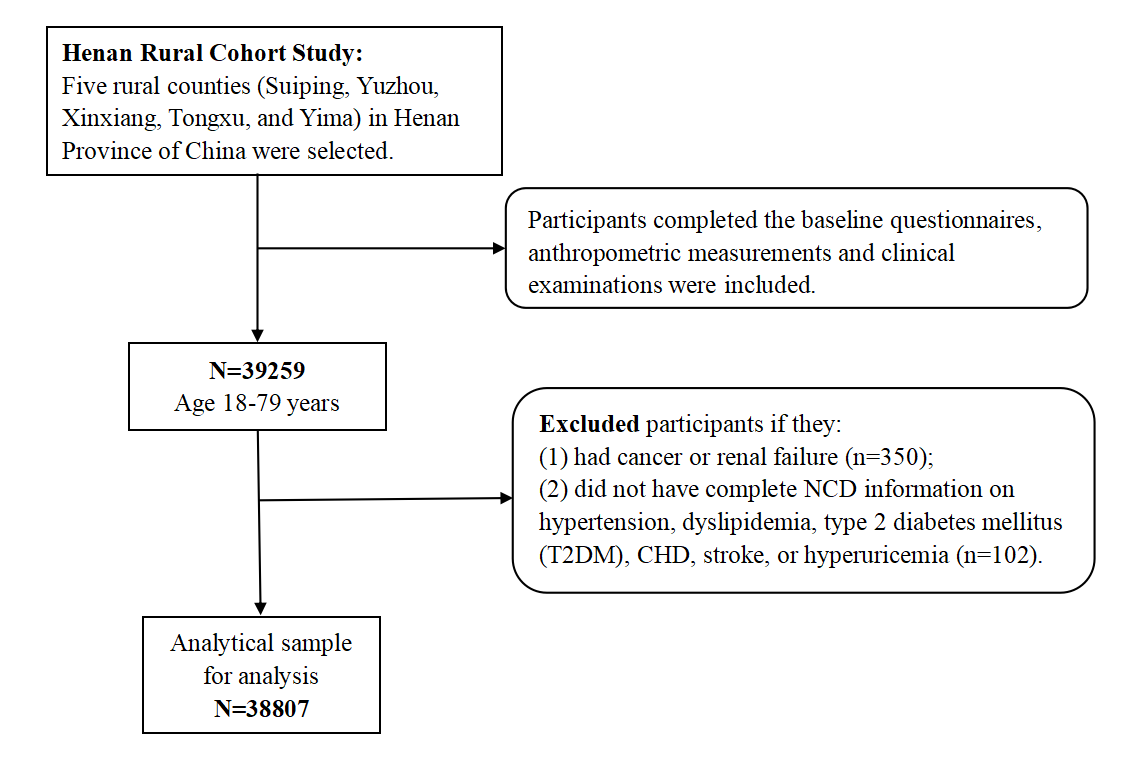

Supplement: Multimedia Appendix 1 [file publichealth_v9i1e43381_app1.png]
