# Supplementary material for: Disease Burden and the Accumulation of Multimorbidity of Noncommunicable Diseases in a Rural Population in Henan, China: Cross-sectional Study
Source: JMIR Public Health Surveill. 2023 May 22;9:e43381. doi: 10.2196/43381 (PMC10242500; doi:10.2196/43381)
Supplement: Multimedia Appendix 3 [file publichealth_v9i1e43381_app3.doc]

**Multimedia Appendix 3.** The association between NCDs pairs based on mean age at diagnosis.

^a^HUA: hyperuricemia.

|  | Prevalence  n (%) | Mean age the diagnosis (years) | DYS^b^ | | HTN^c^ | | CHD^d^ | | T2DM^e^ | | Stroke | |
| --- | --- | --- | --- | --- | --- | --- | --- | --- | --- | --- | --- | --- |
|  |  |  | *OR*^f^ (95%*CI*^g^) | *P* | *OR* (95%*CI*) | *P* | *OR* (95%*CI*) | *P* | *OR* (95%*CI*) | *P* | *OR* (95%*CI*) | *P* |
| **HUA^a^** | 3973 (10.2) | 54.1 | 2.0 (1.9, 2.2) | <.001 | 1.5 (1.4, 1.6) | <.001 | 1.4 (1.2, 1.6) | <.001 | 0.8 (0.7, 0.8) | <.001 | 1.0 (0.9, 1.2) | .74 |
| **DYS** | 14569 (37.5) | 55.1 | —^h^ |  | 1.5 (1.4, 1.6) | <.001 | 1.4 (1.3, 1.6) | <.001 | 2.1 (2.0, 2.3) | <.001 | 1.8 (1.7, 2.0) | <.001 |
| **HTN** | 12692 (32.7) | 55.4 | — |  | — |  | 1.2 (1.1, 1.4) | <.001 | 1.6 (1.5, 1.7) | <.001 | 2.3 (2.1, 2.5) | <.001 |
| **CHD** | 1708 (4.4) | 56.1 | — |  | — |  | — |  | 1.4 (1.2, 1.6) | <.001 | 2.0 (1.8, 2.3) | <.001 |
| **T2DM** | 3664 (9.4) | 56.7 | — |  | — |  | — |  | — |  | 1.7 (1.5, 1.9) | <.001 |
| **Stroke** | 2613 (6.7) | 59.1 | — |  | — |  | — |  | — |  | — |  |

^b^DYS: dyslipidemia.

^c^HTN: hypertension.

^d^CHD: coronary heart disease.

^e^T2DM: Type 2 Diabetes.

^f^OR: odds ratio.

^g^CI:confidence interval.

^h^Not applicable.

Age, body mass index (BMI), gender, educational level, marital status, average monthly individual income, high fat diet, high salt diet, more intake of vegetables and fruits, physical activity, smoking status, drinking status, history of family chronic diseases (hypertension, diabetes, [hyperlipemia](../../../../../Program%20Files%20(x86)/Youdao/Dict/8.9.6.0/resultui/html/index.html#/javascript:;), coronary heart disease, stroke, gout) were adjusted.
