# Supplementary material for: Disease Burden and the Accumulation of Multimorbidity of Noncommunicable Diseases in a Rural Population in Henan, China: Cross-sectional Study
Source: JMIR Public Health Surveill. 2023 May 22;9:e43381. doi: 10.2196/43381 (PMC10242500; doi:10.2196/43381)
Supplement: Multimedia Appendix 4 [file publichealth_v9i1e43381_app4.doc]

Multimedia Appendix 4. The accumulation of NCDs in individuals based on mean age at diagnosis.

|  |  | HTN^a^ | | CHD^b^ | | T2DM^c^ | | Stroke | |
| --- | --- | --- | --- | --- | --- | --- | --- | --- | --- |
|  |  | *OR*^f^ (95%*CI*^g^) | *P* | *OR* (95%*CI*) | *P* | *OR* (95%*CI*) | *P* | *OR* (95%*CI*) | *P* |
| **HUA^d^/DYS^e^** |  |  |  |  |  |  |  |  |  |
| Yes / No |  | 1.3 (1.1, 1.4) | <.001 | 1.4 (1.1, 1.8) | .008 | 0.7 (0.5, 0.8) | .001 | 1.2 (1.0, 1.5) | .08 |
| No / Yes |  | 1.4 (1.3, 1.4) | <.001 | 1.3 (1.2, 1.5) | <.001 | 2.1 (1.9, 2.3) | <.001 | 1.7 (1.6, 1.9) | <.001 |
| Yes / Yes |  | 2.1 (1.9, 2.3) | <.001 | 1.7 (1.4, 2.1) | <.001 | 1.3 (1.2, 1.6) | <.001 | 1.3 (1.1, 1.6) | .004 |
| **HUA/HTN** |  |  |  |  |  |  |  |  |  |
| Yes / No |  | —^h^ |  | 1.5 (1.2, 1.8) | .001 | 0.5 (0.4, 0.7) | <.001 | 0.9 (0.7, 1.2) | .54 |
| No / Yes |  | — |  | 1.2 (1.0, 1.3) | .02 | 1.4 (1.3, 1.6) | <.001 | 2.2 (2.0, 2.4) | <.001 |
| Yes / Yes |  | — |  | 1.4 (1.1, 1.7) | .002 | 1.1 (0.9, 1.2) | .52 | 1.9 (1.6, 2.3) | <.001 |
| **HUA/CHD** |  |  |  |  |  |  |  |  |  |
| Yes / No |  | — |  | — |  | 0.6 (0.6, 0.7) | <.001 | 0.9 (0.8, 1.1) | .35 |
| No / Yes |  | — |  | — |  | 1.3 (1.1, 1.5) | .001 | 2.0 (1.7, 2.3) | <.001 |
| Yes / Yes |  | — |  | — |  | 1.0 (0.7, 1.4) | .83 | 1.3 (0.9, 2.0) | .17 |
| **HUA/T2DM** |  |  |  |  |  |  |  |  |  |
| Yes / No |  | — |  | — |  | — |  | 0.9 (0.8, 1.1) | .33 |
| No / Yes |  | — |  | — |  | — |  | 1.5 (1.3, 1.7) | <.001 |
| Yes / Yes |  | — |  | — |  | — |  | 1.1 (0.8, 1.6) | .56 |
| **DYS/HTN** |  |  |  |  |  |  |  |  |  |
| Yes / No |  | — |  | 1.3 (1.1, 1.5) | <.001 | 2.2 (2.0, 2.4) | <.001 | 1.8 (1.5, 2.0) | <.001 |
| No / Yes |  | — |  | 1.1 (1.0, 1.3) | .20 | 1.5 (1.4, 1.7) | <.001 | 2.3 (2.0, 2.6) | <.001 |
| Yes / Yes |  | — |  | 1.5 (1.3, 1.7) | <.001 | 3.1 (2.8, 3.4) | <.001 | 3.5 (3.1, 4.0) | <.001 |
| **DYS/CHD** |  |  |  |  |  |  |  |  |  |
| Yes / No |  | — |  | — |  | 2.1 (1.9, 2.3) | <.001 | 1.7 (1.5, 1.8) | <.001 |
| No / Yes |  | — |  | — |  | 1.3 (1.1, 1.7) | .01 | 2.1 (1.7, 2.6) | <.001 |
| Yes / Yes |  | — |  | — |  | 2.7 (2.3, 3.3) | <.001 | 2.9 (2.4, 3.5) | <.001 |
| **DYS/T2DM** |  |  |  |  |  |  |  |  |  |
| Yes / No |  | — |  | — |  | — |  | 1.7 (1.5, 1.8) | <.001 |
| No / Yes |  | — |  | — |  | — |  | 1.5 (1.3, 1.9) | <.001 |
| Yes / Yes |  | — |  | — |  | — |  | 2.3 (2.0, 2.7) | <.001 |
| **HTN/CHD** |  |  |  |  |  |  |  |  |  |
| Yes / No |  | — |  | — |  | 1.5 (1.4, 1.6) | <.001 | 2.3 (2.0, 2.5) | <.001 |
| No / Yes |  | — |  | — |  | 1.2 (1.0, 1.6) | .05 | 2.5 (2.1, 3.1) | <.001 |
| Yes / Yes |  | — |  | — |  | 2.0 (1.7, 2.4) | <.001 | 3.4 (2.8, 4.2) | <.001 |
| **HTN/T2DM** |  |  |  |  |  |  |  |  |  |
| Yes / No |  | — |  | — |  | — |  | 2.3 (2.0, 2.5) | <.001 |
| No / Yes |  | — |  | — |  | — |  | 1.8 (1.5, 2.2) | <.001 |
| Yes / Yes |  | — |  | — |  | — |  | 2.9 (2.5, 3.4) | <.001 |
| **CHD/T2DM** |  |  |  |  |  |  |  |  |  |
| Yes / No |  | — |  | — |  | — |  | 1.9 (1.6, 2.2) | <.001 |
| No / Yes |  | — |  | — |  | — |  | 1.4 (1.3, 1.6) | <.001 |
| Yes / Yes |  | — |  | — |  | — |  | 2.7 (2.0, 3.7) | <.001 |

^a^HTN: hypertension.

^b^CHD: coronary heart disease.

^c^T2DM: Type 2 Diabetes.

^d^HUA: hyperuricemia.

^e^DYS: dyslipidemia.

^f^OR: odds ratio.

^g^CI: confidence interval.

^h^Not applicable.

Age, body mass index (BMI), gender, education levels, marital status, average monthly individual income, high fat diet, high salt diet, more intake of vegetables and fruits, physical activity, smoking status, drinking status, history of family chronic diseases (hypertension, diabetes, [hyperlipemia](../../../../../Program%20Files%20(x86)/Youdao/Dict/8.9.6.0/resultui/html/index.html#/javascript:;)**,** coronary heart disease, stroke, gout), and other three NCDs were adjusted.
